# The first pterosaur basihyal, shedding light on the evolution and function of pterosaur hyoid apparatuses

**DOI:** 10.7717/peerj.8292

**Published:** 2020-01-06

**Authors:** Shunxing Jiang, Zhiheng Li, Xin Cheng, Xiaolin Wang

**Affiliations:** 1 Key Laboratory of Vertebrate Evolution and Human Origins, Institute of Vertebrate Paleontology and Paleoanthropology, Chinese Academy of Sciences (CAS), Beijing, China; 2 CAS Center for Excellence in Life and Paleoenvironment, Beijing, China; 3 College of Earth Sciences, Jilin University, Changchun, China; 4 Laboratório de Paleontologia, Universidade Regional do Cariri, Crato, Ceará, Brazil; 5 College of Earth and Planetary Sciences, University of CAS, Beijing, China

**Keywords:** Pterosaurs, Basihyal, Hyoid apparatus, Evolution, Function, Yixian Formation, China

## Abstract

The pterosaur is the first known vertebrate clade to achieve powered flight. Its hyoid apparatus shows a simplification similar to that of birds, although samples of the apparatus are rare, limiting the ability to make an accurate determination. In this study we reveal a new pterosaur specimen, including the first definite basihyal. Through the comparison of pterosaur hyoids, a trend has been discovered for the shortened hyoid relative to the length of the skull, indicating a diminished role of lingual retraction during the evolution of the pterosaur. The new material, possibly from a gallodactylid *Gladocephaloideus*, represents one of the least effective lingual retractions in all pterosaurs. Based on the structure of an elongated ceratobranchial and retroarticular process on mandibles, the function of the Y-shaped istiodactylid tongue bone is similar to those of scavenger crows rather than chameleons, which is consistent with the interpretation of the scavenging behavior of this taxon. More fossil samples are needed for further study on the function of other pterosaur hyoids.

## Introduction

The pterosaur is the first known vertebrate to have obtained the ability of powered flight. The hyoid apparatus of the pterosaur shows a simplification similar to that in birds, in which the muscles play an important role in feeding and respiration (Li, Zhou & Clarke, 2018). The elaboration of the hyoid in pterosaurs is largely unknown because preserved fossils are rare despite widespread descriptions of them (Wellnhofer, 1970; Wellnhofer, 1975; Wellnhofer, 1978; Wellnhofer, 1985; Wellnhofer & Kellner, 1991; Wang & Lü, 2001; Wang et al., 2005; Lü, Xu & Ji, 2008; Padian, 2008a; Padian, 2008b; Dalla Vecchia, 2009; Jiang & Wang, 2011a; Cheng et al., 2012; Wang et al., 2012; Jiang et al., 2014; Lü, 2015).

In general, the ossified bony elements known as the hyoid that are preserved in pterosaur specimens are slender, rod-like ceratobranchial I that vary in length, curvature, and expansion of both tips (Dalla Vecchia, 2009). Williston (1903) considered the proatlas of *Nyctosaurus* (FMNH P 25026) as a basihyal. This interpretation was accepted in the main text but overturned in a note at the end of the monograph by Bennett (2001). Hence, except for two ceratobranchials, the other elements of hyoid apparatus have never been reported in pterosaurs.

A unique type of Y-shaped ceratobranchial has been reported in *Ludodactylus* (SMNK PAL 3828) (Frey, Martill & Buchy, 2003) and *Liaoxipterus* (JLU CAR-0018) (Lü, Xu & Ji, 2008). The extremely elongated and fused anterior parts were used to support a lingual function similar to that in chameleons (Lü, 2015). However, this understanding is challenged based on the new observation of the pterosaur hyoids.

In this study we describe a novel material found in the upper part of the Yixian Formation of Jingangshan locality, where three exceptionally preserved pterosaur and avian embryos were discovered in Jehol Biota (Wang & Zhou, 2004; Ji et al., 2004; Zhou & Zhang, 2004). This new specimen contained the first and well-preserved basihyal that had not been previously reported in any other pterosaur. We are able to approximate the hyoidean evolution in pterosaurs and discuss the function of some special types of hyoid apparatuses based on the newly discovered hyoid apparatus.

## Materials and Methods

The newly discovered specimen (IVPP V 14189) has incomplete mandibular rami with a nearly complete hyoid apparatus and soft tissue impressions. The material was collected from a local farmer in the beginning of the 21st century and was prepared by a professional technician in IVPP. Two istiodactylid specimens were examined under a microscope; one is the holotype of *Nurhachius ignaciobritoi* (IVPP V 13288), whose hyoid was further prepared and first reported here, the other is the holotype of *Liaoxipterus brachyognathus* (JLU CAR-0018).

Further non-invasive observations were made using three-dimensional images of the outer and inner structures of the regions of interest. However, microtomography was not suitable in this case due to the shape of the specimen, which is composed of two broad and relatively thin plates (ca. 30 × 30 ×0.8 cm^3^). Micro X-ray computed laminography (Micro-CL) was conducted in the Key Laboratory of Vertebrate Evolution and Human Origins, Institute of Vertebrate Paleontology and Paleoanthropology, Chinese Academy of Sciences. Image segmentation and visualization were performed using the software VGStudio version 2.2 and Mimix, version 17.0.

## Results

### Description of the new material

**Figure 1:**
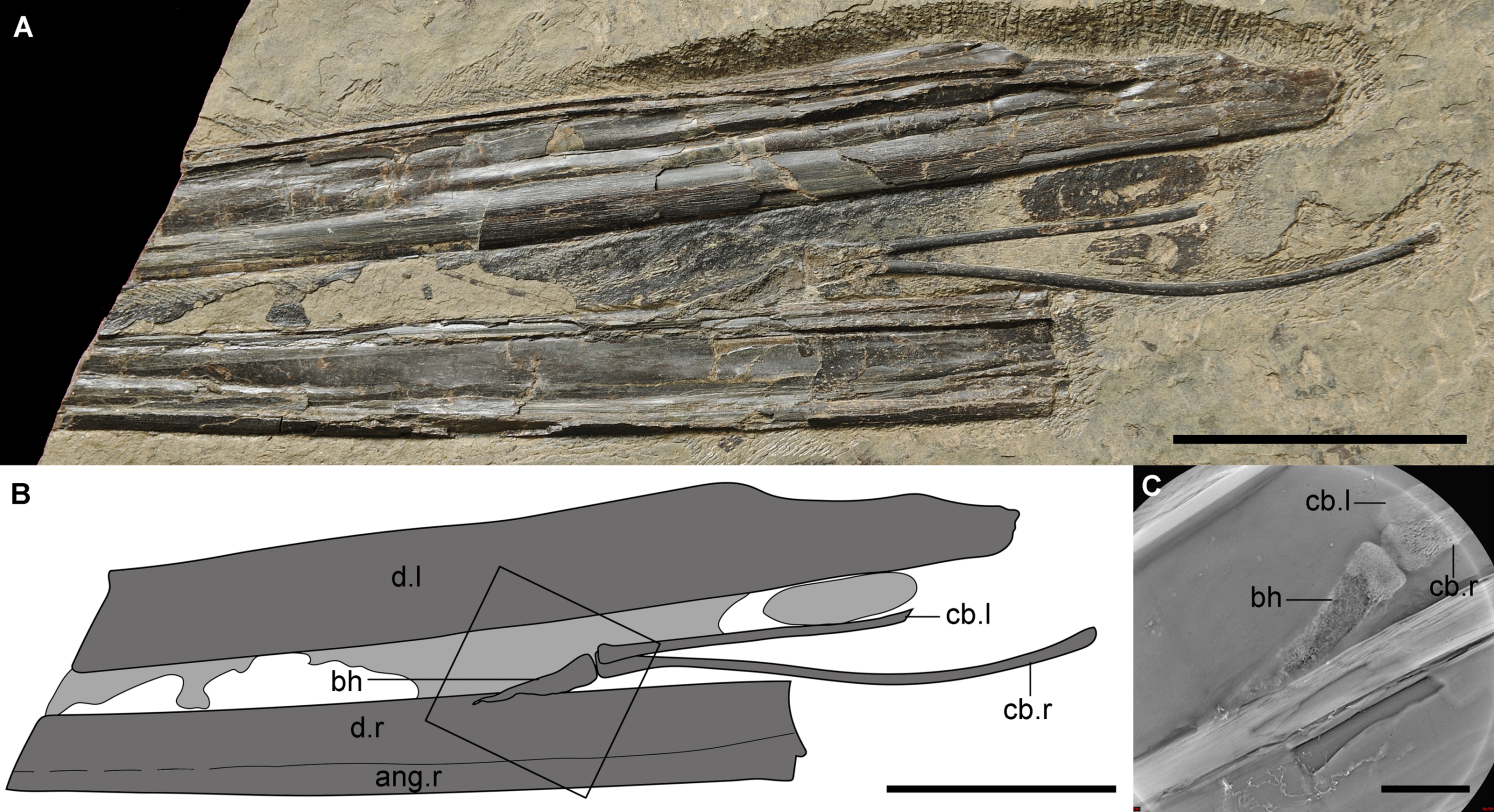
The new material (IVPP V 14189) of a unique hyoid apparatus in pterosaurs from the upper part of Yixian Formation. (A) The photograph (Photo credit: Jie Zhang), scale bar, 50 mm; (B) the line drawing, potential soft tissue shown in light grey, scale bar, 50 mm; (C) the Micro-CL image of basihyal, close-up of the frame in B, scale bar, 10 mm. Abbreviation: ang, angular; bh, basihyal; cb, ceratobranchial; d, dentary; l, left; r, right.

The novel fossil specimen was preserved in a dark grey slab of shale, with black bones and soft tissue impressions (Fig. 1). The preserved mandibular rami did not have any tooth or alveolus, indicating that it was from the posterior portion. The posterior boundaries of both rami were formed by breakage, and the articular was missing. The preserved elements of the mandible were fused quite well without any suture and only the rod-like angular can be distinguished in the medial view of the right ramus. Although a portion of the outer surface of the sample is missing, the rest is smooth with some grooves along the long axis of the mandible on the ventral margins. The preserved lengths of the left and right mandibular rami are 211.0 mm and 175.3 mm, respectively. The depth of the left ramus is 22.4 mm without apparent changes, while the right ramus slightly tapers, from 21.6 mm to 17.3 mm. The different depths of the two sides are likely caused by the different portion of the ramus or by the taphonomy, with the latter explanation preferred.

The hyoid apparatus includes a pair of ceratobranchial I and a middle-line element, the basihyal. The ceratobranchials are two thin, rod-like bones. The right one was complete with an expanded anterior tip (4.6 mm width) that was larger than the posterior expansion (3.0 mm width). According to the smaller posterior expansion, the possible existence of epibranchials was inferred, which has not been reported in any pterosaur; epibranchials may be similar to the cartilage seen in some modern birds. The length of the right ceratobranchial between both tips was 106.2 mm, and the width was 2.1 mm. There were shallow grooves, observable by microscope, found on the entire surface but no origin or insertion of any muscles were detected. The right ceratobranchial was slightly curved upward at the middle portion. The basihyal was an elongated triangular element, with a maximal length of 29.2 mm and a width of 6.1 mm at the base. The basihyal seemed to be twisted, indicating that it was potentially cartilaginous, or possibly cartilage that was not fully ossified, which was supported by Micro-CL imaging (Fig. 1C). The posterior aspect of the basihyal was flat as the anterior ones of ceratobranchials, inferring that the articulations between the basihyal and ceratobranchials were not flexible. The anterior end of the basihyal was pointed, inferring that the paraglossal was nonexistent, which is common in neognath birds and complicated in paleognaths (Li, Zhou & Clarke, 2018). This phenomenon has only been reported in one taxon of dinosaur (Hill et al., 2015), but was not preserved in extant crocodilians and turtles (Li & Clarke, 2015).

### Identifying the new material

The mandibular rami were partially preserved, which was identified as the toothless caudal segment. Two pterosaur taxa have been reported so far in the Jingangshan locality, namely the gallodactylid *Gladocephaloideus jingangshanensis* (Lü et al., 2012), and a questionable *Yixianopterus jingangshanensis*, in which most of the cranial bone was artificially reconstructed (Lü et al., 2006). The new material would be best assigned to *Gladocephaloideus* rather than *Yixianopterus* due to the location in which it was found. The depth of the posterior end of the new material was four times large as that of the holotype of *Gladocephaloideus*, which indicates that the sizes are quite different between these two specimens. The holotype was considered to be an adult individual (Lü et al., 2012), but was later reconsidered to be a late juvenile or sub-adult individual (Lü, Kundrát & Shen, 2016). It is reasonable to believe that the new material is from an adult *G. jingangshanensis* and is quite larger than the holotype.

Unpublished collections from the same location housed at IVPP (IVPP V 12695A&B, V 13335, V 14235, V 14427, and V 26616) reveal some pterosaurs with different characteristics from *Gladocephaloideus*, suggesting the diversity of pterosaurs in this vicinity. The new material can be excluded as being from istiodactylids and *Guidraco* because of the different shapes of the hyoids. The length/depth ratio of the preserved toothless mandibular rami in the new material was 9.4, and the accurate value should be even larger because of the incompleteness of the sample, while the ratios of tapejarids, chaoyangopterids, anurognathid, boreopterids, anhanguerids, and some other pteranodontoids (*sensu* Kellner, 2003) found in Jehol Biota were less than 10; only ctenochasmatid and gallodactylid pterosaurs have a greater ratio than this value. The ratios are 10.6 and 10.2 in *Gegepterus* and *Forfexopterus*, respectively (Wang et al., 2007; Jiang et al., 2016). These are the only two published ctenochasmatids with nearly complete mandibles from the Jehol Biota. The incompleteness of the new sample and the slightly larger ratios in ctenochasmatids lead us to believe that *G*. *jingangshanensis*, a gallodactylid pterosaur, is the best available interpretation.

## Discussion

### The hyoidean evolution in pterosaurs

**Figure 2:**
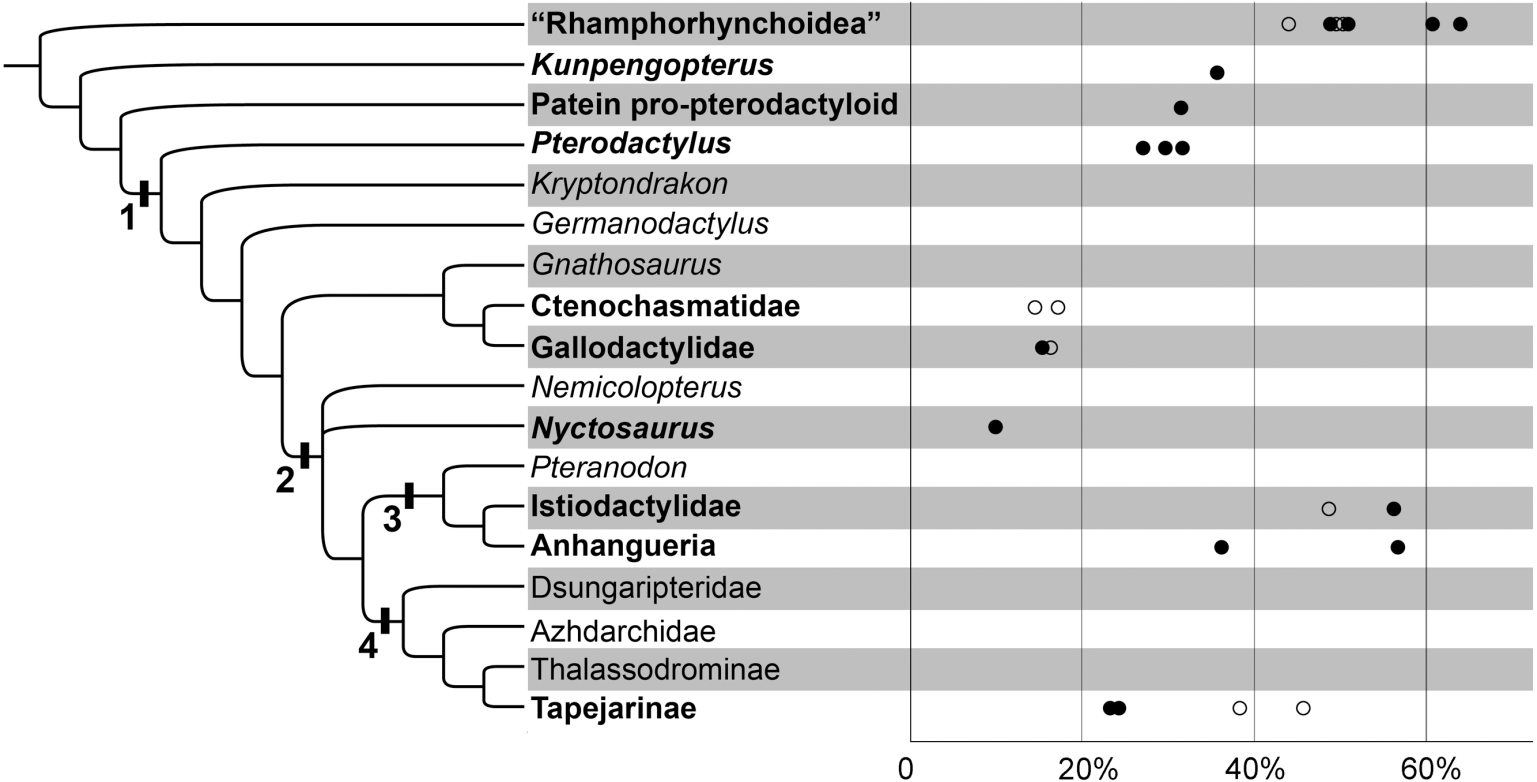
The variability of the ceratobranchial relative to the skull in pterosaurs. The phylogenetic tree after Wang et al., 2017. 1. Pterodactyloidea; 2. Dsungaripteroidea; 3. Pteranodontoidea; 4. Tapejaroidea. The group of pterosaurs with hyoids are in bold. The horizontal axis indicates the ceratobranchial/skull length ratio; the circles indicate the estimated ones; details can be seen in Table 1.

Several phylogenetic analyses have been carried out on pterosaur to date (Kellner, 2003; Unwin, 2003; Wang et al., 2009; Lü et al., 2010; Andres, Clark & Xu, 2014; Codorniú et al., 2016; Wang et al., 2017; Vidovic & Martill, 2017), although no consensus on the evolution of the pterosaur has been reached. The evolution from long-tailed taxa to short-tailed one is commonly accepted; however, this study does not address the comparisons made between the differences in published phylogenetic results. The phylogenetic results obtained from previously conducted research is included in this study done by some authors before is chosen here (Wang et al., 2017), which was also on some previous studies (Kellner, 2003; Wang et al., 2009). Some modifications were made for the discussion of hyoidean evolution (Fig. 2).

**Table 1:** The ceratobranchials/skull length ratios in Pterosauria.

Taxa	Ceratobranchial/ skull length ratio	Sources	Comments
*Eudimorphodon ranzii* (MCSNB 2888)	>30%	Zambelli, 1973; Paganoni, 2003	incomplete hyoid
*Austriadactylus cristatus* (SMNS 56342)	∼44%	Dalla Vecchia et al., 2002	posterior most missing
*Dorygnathus banthensis* (SMNS 50914)	>34.0%	Padian, 2008a	posterior part missing
*Dorygnathus banthensis* (SMNS 50702)	63.8%	Padian, 2008a	
*Dorygnathus banthensis* (SMNS 51827)	>30.5%	Padian, 2008a	anterior and posterior parts missing
*Scaphognathus crassirostris* (SMNS 59395)	50.8%	Bennett, 2014	
*Scaphognathus crassirostris* (GPIB 1304)	∼49.4%	Bennett, 2014	anterior most missing
*Jianchangnathus robustodens* (IVPP V 16866)	>37.6%	Cheng et al., 2012	anterior part missing
*Fenghuangopterus lii* (CYGB-0037)	60.70%	Lü et al., 2010	
*Rhamphorhynchus muensteri* (BSP AS VI 34)	>36.3%	Wellnhofer, 1975	anterior part missing
*Rhamphorhynchus muensteri* (BSP 1927 I 36)	∼50.5%	Wellnhofer, 1975	anterior most missing
*Darwinopterus robustodens* (HGM 41HIII-0309A)	49.1%	Lü et al., 2011	
*Darwinopterus modularis* (ZNHM M8782)	>25%	Lü et al., 2010	anterior part missing
*Kunpengopterus sinensis* (IVPP V 23674)	35.7%	Cheng et al., 2017	
Painten pro-pterodactyloid	31.4%	Tischlinger & Frey, 2013	
*Pterodactylus antiquus* (BSP 1883 XVI 1 / MCZ 1505)	29.6%	Wellnhofer, 1970; Bennett, 2013	
*Pterodactylus kochi* (BSP 1975 I 221)	31.6%	This paper	
*Pterodactylus* (JME-SOS 4008)	27.0%	This paper	
*Gegepterus changae* (IVPP V 11981)	∼14.5%	Wang et al., 2007	skull length estimated
*Pterofiltrus qiui* (IVPP V 12339)	∼17.2%	Jiang & Wang, 2011b	anterior most missing
*Feilongus youngi* (IVPP V 12539)	15.4%	Wang et al., 2005	
*Gladocephaloideus jingangshanensis* (IG-CAGS-08-07)	∼16.4%	Lü et al., 2012	anterior most missing
*Nyctosaurus* (FMNH P 25026)	10%	Williston, 1902; Williston, 1903; Wellnhofer, 1978	
*Nurhachius ignaciobritoi* (IVPP V 13288)	>39.7%	Wang et al., 2005	posterior part missing
*Liaoxipterus brachyognathus* (JLU CAR-0018)	∼48.6%	Lü, Xu & Ji, 2008	skull length estimated from similar taxa
Istiodactylid (BHGL-036)	56.2%	This paper	
*Ludodactylus sibbicki* (SMNK PAL 3828)	56.6%	Frey, Martill & Buchy, 2003	
*Guidraco venato* (IVPP V 17083)	>37.4%	Wang et al., 2012	anterior part missing
*Haopterus gracilis* (IVPP V 11726)	36.1%	Wang & Lü, 2001	
*Anhanguera araripensis* (BSP 1982 I 89)	>22%	Wellnhofer, 1985	posterior part missing
*Ikrandraco avatar* (IVPP V 18406)	>16.9	Wang et al., 2014	posterior part missing
*Sinopterus* sp. (IVPP V 20249)	23.3%	This paper	
*Sinopterus* sp. (IVPP V 26617)	24.3%	This paper	
*Europejara olcadesorum* (MCCM-LH 9413)	∼45.8%	Vullo et al., 2012	nearly complete
*Tapejara wellnhoferi* (AMNH 24440)	∼38.3%	Wellnhofer & Kellner, 1991	nearly complete

In most birds, the hyoid apparatus includes two rod-like ceratobranchials, two partially cartilaginous curved epibranchials, a small middle-line basihyal and urohyal, and a pair of cartilaginous paraglossa (Homberger, 2017). In pterosaurs, the ceratobranchials are similar; the basihyal is only preserved in the new specimen, and the other elements are absent. Based on the anterior and posterior expansions of the ceratobranchial tips, the basihyal should be more common than the condition in preserved fossils and the epibranchials likely existed in some pterosaur taxa. There is no evidence for the urohyal or paraglossal.

**Figure 3:**
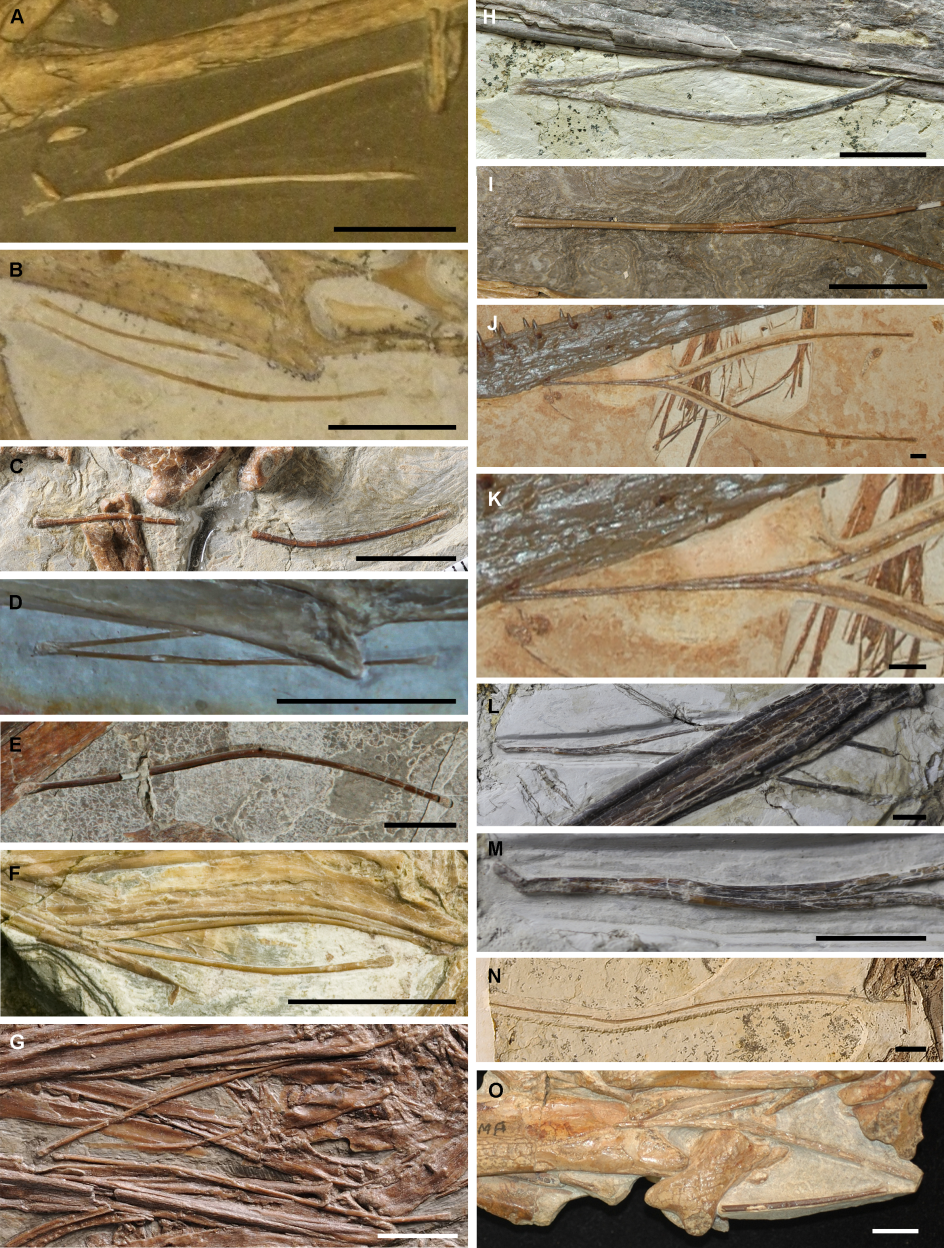
Hyoid apparatuses in pterosaurs. (A) *Dorygnathus banthensis* SMNS 50914 (Photo credit: Taissa Rodrigues); (B) *Scaphognathus crassirostris* SMNS 59395 (Photo credit: Taissa Rodrigues); (C) *Kunpengopterus sinensis*, IVPP V 23674; (D) *Pterodatylus*, JME-SOS 4008 (Photo credit: Rui Qiu); (E) *Feilongus youngi*, IVPP V 12539; (F) *Gegepterus changae*, IVPP V 11972; (G) *Haopterus gracillis*, IVPP V 11726; (H) *Ikrandraco avatar*, IVPP V 18199; (I) *Liaoxipterus brachyognathus*, JLU CAR-0018, close-up of the anterior part of hyoid; (J) & (K) *Ludodactylus sibbicki*, SMNK PAL 3828 (Photo credit: Alexander. W.A. Kellner), K, close-up of the anterior part of hyoid; (L) & (M) *Nurhachius ignaciobritoi*, IVPP V 13288, M, close-up of the anterior part of hyoid; (N) *Guidraco venator*, IVPP V 17033; O, *Tapejara wellnhoferi*, AMNH 24440 (Photo credit: Alexander W.A. Kellner). The anterior ends to the left side, and E, G, J, K, N, & O shown inverted. Scale bars, 10 mm.

The hyoids of primitive non-pterodactyloids only include the preserved ceratobranchials; this rod-like element is slender and quite long relative to the skull length (Figs. 2; 3A & 3B; Table 1). The ceratobranchial/skull length ratios are 63.8%, 50.8%, and 49.1% in *Dorygnathus banthensis* (SMNS 50702) (Padian, 2008b), *Scaphognathus crassirostris* (SMNS 59395) (Bennett, 2014), and *Darwinopterus robustodens* (HGM 41HIII-0309A) (Lü et al., 2011), respectively. A similar condition was found in *Austriadactylus cristatus* (SMNS 56342) and *Rhamphorhynchus muensteri* (BSP 1927 I 36), although the ceratobranchials are slightly incomplete. The ceratobranchial/skull length ratio is similar to most extant reptiles (Li & Clarke, 2015). In some non-pterodactyloids, the anterior end expanded, referring the presence of the basihyal, represented by *Carniadactylus rosenfieldi* (MFSN 1797) (Elsheikh & Al-Zahaby, 2014), *Scaphognathus crassirostris* (SMNS 59395) (Bennett, 2014), and *Darwinopterus robustodens* (HGM 41HIII-0309A) (Lü et al., 2011). The basihyal probably appeared in the primitive pterosaur from the Late Triassic, although this newly discovered sample from the late Early Cretaceous period is the only basihyal in pterosaurs and it is absent from the fossil records due to its cartilaginous bone tissue. The ceratobranchials are gently curved, except in some species in which it is nearly straight. For example, three specimens of *Dorygnathus banthensis* are straight without any bend (Fig. 3A) (Padian, 2008b), nearly excluding the influence of the taphonomy.

The ceratobranchial/skull length ratios have a decreasing trend from long-tailed pterosaurs to short-tailed Pterodactyloidea (Fig. 2; Table 1). In *Kunpengopterus* (Fig. 3C), Paiten pro-pterodactyloid, and *Pterodactylus* (Fig. 3D), the ceratobranchials shortens to approximately 30% of the skull length (Cheng et al., 2017; Tischlinger & Frey, 2013; Bennett, 2013; Wellnhofer, 1970), while the ratios dropped to less than 20% in the gallodactylids *Gladocephaloideus* (Lü et al., 2012) and *Feilongus* (Fig. 3E) (Wang et al., 2005), as well as in ctenochasmatid pterosaurs, such as *Gegepterus* (Fig. 3F) (Wang et al., 2007; Jiang & Wang, 2011a), and *Pterofiltrus* (Jiang & Wang, 2011b).

Pteranodontoid (*sensu* Kellner, 2003) pterosaurs display the unexpanded anterior ends of ceratobranchials, suggesting the absence of the basihyal in Istiodactylidae and Anhangueria (Figs. 3H–3N). The decreasing trend was reversed in istiodactylids and *Ludodactylus sibbicki* (SMNK PAL 3828), whose ceratobranchials are quite elongated and slightly tapered forward (Frey, Martill & Buchy, 2003; Lü, Xu & Ji, 2008). The anterior fusion of ceratobranchials was considered to be the lingual process in *Ludodactylus* and *Liaoxipterus* (Lü, 2015). The unfused two ceratobranchials can be identified by the observation of the holotype of *Liaoxipterus* (Fig. 3I), and the condition is also seen in the photos of *Ludodactylus* (Figs. 3J & 3K). The further prepared holotype of *Nurhachius* (IVPP V 13288), which is the first specimen of istiodactylids from China, also shows the unfused ceratobranchials with the anterior portions close to each other (Figs. 3L &  3M). Therefore, no fusion of the ceratobranchials existed in pterosaurs, contrary to what has been described in earlier literature (Lü, 2015). *Guidraco* is closely related to *Ludodactylus* and has only one elongated ceratobranchial (Fig. 3N) (Wang et al., 2012), which may also have the Y-shaped ceratobranchials.

The information on hyoids in Tapejaroidea (*sensu* (Kellner, 2003) is limited to three taxa in Tapejaridae: *Europejara*, *Sinopterus*, and *Tapejara* (Fig. 3O). These three taxa, in a monophyletic group, shared a similar shape of ceratobranchials, but with varieties in their lengths relative to their skull (Fig. 2; Table 1). *Europejara* (MCCM-LH 9413) is the greatest at 45.8% (Vullo et al., 2012) and *Sinopterus* is the least at 25%.

### The function of some pterosaur hyoids

**Figure 4:**
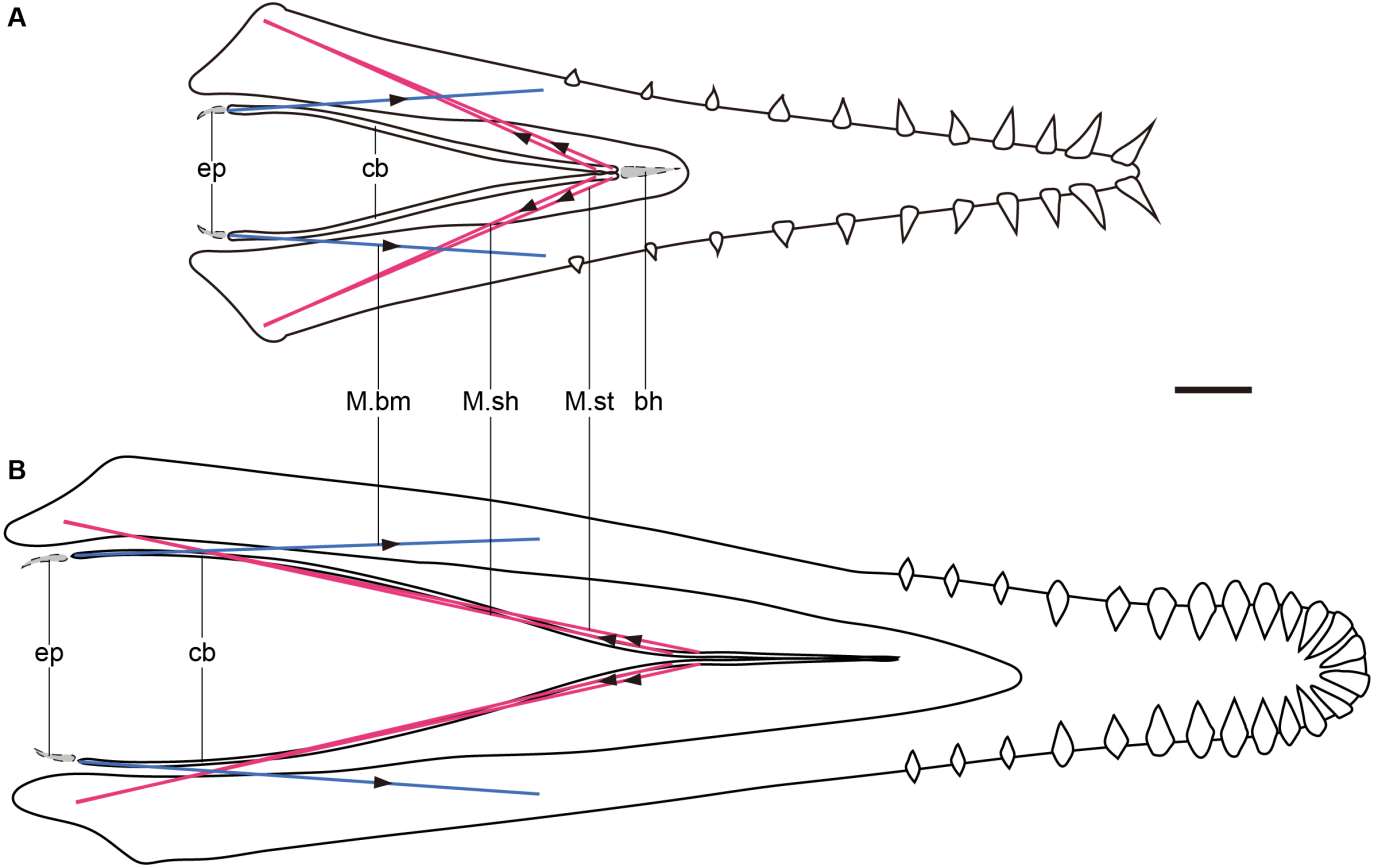
Graphic models of lingual muscles of *Haopterus* and *Liaoxipterus* in dorsal view. (A) *Haopterus*; (B) *Liaoxipterus*. Red lines indicate the inferred M. sh and M. st, and blue lines indicate the inferred M.bm. The arrows indicate direction of movements and forces. The assumed epibranchials and basihyal are shown in grey within the dashed lines. Abbreviation: bh, basihyal; cb, ceratobranchial; ep, epibranchial; M.bm, *M. branchiomandibularis*; M. sh, *M. serpihyoideus*; M. st, *M. stylohyoideus*. Scale bar, 10 mm.

There is a trend in the Pterosauria showing that the ceratobranchials are shortened relative to their skull length (Fig. 2, Table 1). The structure of the pterosaur hyoid apparatus is simplified as in birds, with elongated ceratobranchials. In birds, there are a pair of muscles that control the protraction and retraction of the tongue. *M. branchiomandibularis* (M.bm) originates from the mandibles and is inserted on the posterior end of ceratobranchials and the anterior end of the epibranchials, which active in tongue retraction (Homberger & Meyers, 1989). *M. serpihyoideus* (M.sh) and *M. stylohyoideus* (M.st) originate from the retroarticular process of the mandible and insert on the anterior of the basihyal and ceratobranchials, rarely on the paraglossal, and it can protract the tongue (Homberger & Meyers, 1989). It is assumed that pterosaurs had similar lingual muscles to those of birds, with a similar lifestyle and a simplified hyoid apparatus, which is a reasonable assumption as birds are the closest extant group to the pterosaur in most phylogenetic results (Witton, 2013). The M.bm is inserted on the posterior end of the ceratobranchial whether epibranchials are present in the pterosaur or not. If the origin of the M.bm does not change, the length of M.bm will be the same (Fig. 4), indicating that protraction is maintained when the ceratobranchials shorten. However, the lengths of the M.sh and M.st were shortened (Fig. 4), indicating that there was a decreased ability to retract the tongue and a lingual inability to transport food during the evolution of the pterosaur. The new material, which is presumed to be gallodactylid *Gladocephaloideus*, should have an elongated upper and lower jaw, with a short tooth row (Lü et al., 2012). The length of the ceratobranchials in the new material was one of the shortest relative to the skull in all known pterosaurs and the ability of lingual transport of food may have been the least effective.

The elongated Y-shaped hyoid apparatuses (Fig. 4B), represented by istiodactylids, reversed the trend of the length decrease, which suggests the essential function of the tongue. Lü (2015) considered the fused (although actually unfused) anterior part of ceratobranchials as the lingual process reported in chameleons, along which the tongue slides during launch. In the prey processing of chameleons, the soft tissue of the tongue projects out of the mouth along with the hyoid element; the entoglossal process moves forward relative to the ceratobranchials and ceratohyals of the hyoid apparatus (Herrel et al., 2001). In istiodactylids, the anterior process, made up of the anterior of ceratobranchials, cannot move forward relative to the rest of the ceratobranchials, indicating the limited anteroposterior movement of the tongue. Therefore, this Y-shaped hyoid is different from that of chameleons, and istiodactylids were likely not insectivorous as was suggested in the previous literature (Lü, 2015). However, this specialized type of hyoid represents some other function of their tongues in feeding or respiration. A careful examination of this Y-shaped hyoid reveals a slight expansion in the corner of the bend in some specimens, which might be near the posterior end of the adhered anterior parts. When compared with avian muscles, this expansion is probably the insertion of M.st, which originates from the lateral and ventral aspects of the caudal mandible, primarily on the retroarticular process (Homberger & Meyers, 1989; Li & Clarke, 2016). This process is more elongated in istiodactylids than that of most other pterosaurs (Witton, 2012). The elongation of the ceratobranchials and the enlarged area for the muscular attachment indicates the development of this muscle, as well as the enhanced ability to retract the tongue, relative to powerful food transport. In some extant crows (*Corvus*), which are scavengers, the anterior part of the tongue can protrude out of the oropharyngeal cavity and transport food during the retraction, which is an adaption necessary for the food collection (Igwebuike & Eze, 2010; Elsheikh & Al-Zahaby, 2014). The tongue of istiodactylids may not have needed to protrude out like that of crows, because they may have used their diamond-shaped teeth to cut the food into pieces. The only elongated ceratobranchials also provide the tongue firmness as well as the bone function in crows. Therefore, istiodactylids were probably scavengers, as suggested in the previous literature (Witton, 2012). *Ludodactylus* also had the Y-shaped hyoid, which might have similar functions to istiodactylids. However, the presumed Y-shaped hyoid pterosaur, *Guidraco*, has exaggerated anterior teeth unfitting for a scavenger and the hyoid function requires further discussion.

## Conclusion

The new fossil material revealed a novel type of hyoid apparatus and demonstrations the first basihyal in pterosaurs, revealed the hyoid morphology previously unseen. The hyoids in pterosaurs have a trend to shorten relative to the length of skulls, indicating the diminished role in lingual transport of food from the non-pterodactyloids to the pterodactyloids. The new gallodactylid, *Gladocephaloideus,* represents one of the least effective lingual retractions in all pterosaurs. Based on the elongated ceratobranchials and shape of the retroarticular process, the function of the Y-shaped istiodactylid tongue is unlike that of chameleons but closer to that of scavenging crows, which is consistent with the interpretation of the scavenging behavior in pterosaurs. More fossil samples are needed for further study on the function of other hyoids.

## Institutional abbreviations

AMNH: American Museum of Natural History, New York, USA
BHGL: Beipiao Pterosaur Museum of China, Chaoyang, China
BSP: Bayerische Staatssammlung für Paläontologie und historische Geologie, Munich, Germany
CYGB: Chaoyang Bird Fossil National Geopark, China
FMNH: Field Museum of Natural History, Chicago, USA
GBIP: Geologische Institut für Paläontologie der Universität Bonn, Germany
HGM: Henan Geological Museum, Zhengzhou, China
HM: Hami Museum, Hami, China
IG-CAGS: Institute of Geology, Chinese Academy of Geological Sciences, Beijing, China
IVPP: Institute of Vertebrate Paleontology and Paleoanthropology, Chinese Academy of Sciences, Beijing, China
JLU: Jilin University, Changchun, China
JME-SOS: JuraMuseum (Solnhofen Sammlung), Eichstätt, Germany
MCCM-LH: Las Hoyas collection of the Museo de las Ciencias de Castilla-La Mancha, Cuenca, Spain
MCSNB: Museo Civico di Scienze Naturali di Bergamo, Italy
MCZ: Museum of Comparative Zoology, Harvard University, Cambridge, USA
MFSN: Museo Friulano di Storia Naturale, Udine, Italy
SMNK: Staatliches Museum für Naturkunde Karlsruhe, Germany
SMNS: Staatliches Museum für Naturkunde Stuttgart, Germany
ZNHM: Zhejiang Museum of Natural History, Hangzhou, China

## Additional Information and Declarations

### Data Availability

The following information was supplied regarding data availability: The raw data is available in Table 1. The main specimens: the new material (IVPP V 14189) and *Nurhachius ignaciobritoi* (IVPP V 13288) are housed at Institute of Vertebrate Paleontology and Paleoanthropology, Chinese Academy of Sciences, Beijing, China. *Liaoxipterus brachyognathus* (JLU CAR-0018) is housed at Jilin University, Changchun, China. The other specimens we examined are as follows: - unpublished specimens: IVPP V 12695A&B, V 13335, V 14235, V 14427, and V 26616. - *Dorygnathus banthensis* (SMNS 50702, SMNS 50914, SMNS 51827) - *Scaphognathus crassirostris* (SMNS 59395, GPIN 1304) - *Darwinopterus robustodens* (HGM 41HIII-0309A) - *Austriadactylus cristatus* (SMNS 56342) - *Rhamphorhynchus muensteri* (BSP 1927 I 36, BSP 1927 I 36) - *Carniadactylus rosenfieldi* (MFSN 1797) - *Kunpengopterus sinensis* (IVPP V 23674) - Painten pro-pterodactyloid Painten specimen (Tischlinger & Frey, 2013) - *Pterodactylus* (JME-SOS 4008) - *Feilongus youngi* (IVPP V 12539) - *Gegepterus changae* (IVPP V 11972, IVPP V 11981) - *Pterofiltrus qiui* (IVPP V 12339) - *Forfexopterus jeholensis* (HM V20) - *Ludodactylus sibbicki* (SMNK PAL 3828) - *Guidraco venator* (IVPP V 17083) - *Tapejara wellnhoferi* (AMNH 24440) - *Gladocephaloideus jingangshanensis* (IG-CAGS-08-07) - *Nyctosaurus* (FMNH P 25026) - *Haopterus gracilis* (IVPP V 11726) - *Anhanguera araripensis* (BSP 1982 I 89) - *Ikrandraco avatar* (IVPP V 18406) - *Sinopterus* sp. (IVPP V 20249) - *Sinopterus* sp. (IVPP V 26617) - *Europejara olcadesorum* (MCCM-LH 941) - *Eudimorphodon ranzii* (MCSNB 2888) - *Jianchangnathus robustodens* (IVPP V 16866) - *Fenghuangopterus lii* (CYGB-0037) - *Darwinopterus modularis* (ZNHM M8782) - *Pterodactylus antiquus* (BSP 1883 XVI 1 / MCZ 1505) - *Pterodactylus kochi* (BSP 1975 I 221) - *Istiodactylid* (BHGL-036)

## References

[ref-1] Andres B, Clark JM, Xu X (2014). The earliest pterodactyloid and the origin of the group. Current Biology.

[ref-2] Bennett SC (2001). The osteology and functional morphology of the Late Cretaceous pterosaur *Pteranodon*. Palaeontographica Abteilung A.

[ref-3] Bennett SC (2013). New information on body size and cranial display structures of *Pterodactylus antiquus*, with a revision of the genus. Paläontologische Zeitschrift.

[ref-4] Bennett SC (2014). A new specimen of the pterosaur *Scaphognathus crassirostris*, with comments on constraint of cervical vertebrae number in pterosaurs. Neues Jahrbuch für Geologie und Paläontologie—Abhandlungen.

[ref-5] Cheng X, Jiang S, Wang X, Kellner AWA (2017). New anatomical information of the wukongopterid *Kunpengopterus sinensis* Wang et al. 2010 based on a new specimen. PeerJ.

[ref-6] Cheng X, Wang X, Jiang S, Kellner AWA (2012). A new scaphognathid pterosaur from western Liaoning, China. Historical Biology.

[ref-7] Codorniú L, Paulina-Carabajal A, Pol D, Unwin DM, Rauhut OWM (2016). A Jurassic pterosaur from Patagonia and the origin of the pterodactyloid neurocranium. PeerJ.

[ref-8] Dalla Vecchia FM (2009). Anatomy and systematics of the pterosaur *Carniadactylus* gen. n. *rosenfeldi* (Dalla Vecchia, 1995). Rivista Italiana di Paleontologia e Stratigrafia (Research In Paleontology and Stratigraphy).

[ref-9] Dalla Vecchia FM, Wild R, Hopf H, Reitner J (2002). A crested rhamphorhynchoid pterosaur from the Late Triassic of Austria. Journal of Vertebrate Paleontology.

[ref-10] Elsheikh EH, Al-Zahaby SA (2014). Light and scanning electron microscopical studies of the tongue in the hooded crow (Aves: *Corvus corone cornix*). The Journal of Basic & Applied Zoology.

[ref-11] Frey E, Martill DM, Buchy M-C, Buffetaut E, Mazin JM (2003). A new crested ornithocheirid from the Lower Cretaceous of northeastern Brazil and the unusual death of an unusual pterosaur. Evolution and palaeobiology of pterosaurs.

[ref-12] Herrel A, Meyers JJ, Nishikawa KC, De Vree F (2001). Morphology and histochemistry of the hyolingual apparatus in chameleons. Journal of Morphology.

[ref-13] Hill RV, D’Emic MD, Bever GS, Norell MA (2015). A complex hyobranchial apparatus in a Cretaceous dinosaur and the antiquity of avian paraglossalia. Zoological Journal of the Linnean Society.

[ref-14] Homberger DG, Maina JN (2017). The avian lingual and laryngeal apparatus within the context of the head and jaw apparatus, with comparisons to the mammalian condition: functional morphology and biomechanics of evaporative cooling, feeding, drinking, and vocalization. The biology of the avian respiratory system, evolution, development, structure and function.

[ref-15] Homberger DG, Meyers RA (1989). Morphology of the lingual apparatus of the domestic chicken, *Gallus gallus*, with special attention to the structure of the fasciae. American Journal of Anatomy.

[ref-16] Igwebuike UM, Eze UU (2010). Anatomy of the oropharynx and tongue of the African pied crow (*Corvus albus*). Veterinarski Arhiv.

[ref-17] Ji Q, Ji S, Cheng Y, You H, Lü J, Liu Y, Yuan C (2004). Pterosaur egg with a leathery shell. Nature.

[ref-18] Jiang S, Cheng X, Ma Y, Wang X (2016). A new archaeopterodactyloid pterosaur from the Jiufotang formation of western Liaoning, China, with a comparison of sterna in Pterodactylomorpha. Journal of Vertebrate Paleontology.

[ref-19] Jiang S, Wang X (2011a). Important features of *Gegepterus changae* (Pterosauria: Archaeopterodactyloidea, Ctenochasmatidae) from a new specimen. Vertebrata Palasiatica.

[ref-20] Jiang S, Wang X (2011b). A new ctenochasmatid pterosaur from the Lower Cretaceous, western Liaoning, China. Anais da Academia Brasileira de Ciencias.

[ref-21] Jiang S, Wang X, Meng X, Cheng X (2014). A new boreopterid pterosaur from the Lower Cretaceous of western Liaoning, China, with a reassessment of the phylogenetic relationships of the Boreopteridae. Journal of Paleontology.

[ref-22] Kellner AWA, Buffetaut E, Mazin JM (2003). Pterosaur phylogeny and comments on the evolutionary history of the group. Evolution and palaeonbiology of pterosaurs.

[ref-23] Li Z, Clarke JA (2015). New insight into the anatomy of the hyolingual apparatus of *Alligator mississippiensis* and implications for reconstructing feeding in extinct archosaurs. Journal of Anatomy.

[ref-24] Li Z, Clarke JA (2016). The craniolingual morphology of waterfowl (Aves, Anseriformes) and its relationship with feeding mode revealed through contrast-enhanced X-ray computed tomography and 2D morphometrics. Evolutionary Biology.

[ref-25] Li Z, Zhou Z, Clarke JA (2018). Convergent evolution of a mobile bony tongue in flighted dinosaurs and pterosaurs. PLOS ONE.

[ref-26] Lü J (2015). The hyoid apparatus of *Liaoxipterus brachycephalus* (Pterosauria) and its implications for food-catching behavior. Acta Geoscientica Sinica.

[ref-27] Lü J, Ji Q, Wei X, Liu Y (2012). A new ctenochasmatoid pterosaur from the Early Cretaceous Yixian Formation of western Liaoning, China. Cretaceous Research.

[ref-28] Lü J, Ji S, Yuan C, Gao Y, Sun Z, Ji Q, Lü J, Kobayashi Y, Huang D, Lee Y-N (2006). New pterodactyloid pterosaur from the Lower Cretaceous Yixian Formation of western Liaoning.

[ref-29] Lü J, Kundrát M, Shen C (2016). New material of the pterosaur *Gladocephaloideus* Lü, et al. 2012 from the Early Cretaceous of Liaoning Province, China, with comments on its systematic position. PLOS ONE.

[ref-30] Lü J, Unwin DM, Jin X, Liu Y, Ji Q (2010). Evidence for modular evolution in a long-tailed pterosaur with a pterodactyloid skull. Proceedings of the Royal Society B-Biological Sciences.

[ref-31] Lü J, Xu L, Chang H, Zhang X (2011). A new darwinopterid pterosaur from the Middle Jurassic of western Liaoning, northeastern China and its ecological implications. Acta Geologica Sinica (English Edition).

[ref-32] Lü J, Xu L, Ji Q (2008). Restudy of *Liaoxipterus* (Istiodactylidae: Pterosauria), with comments on the Chinese istiodactylid pterosaurs. Zitteliana Reihe B.

[ref-33] Padian K (2008a). The Early Jurassic pterosaur *Campyloganthoides* (Strand, 1928). Special Papers in Palaeontology.

[ref-34] Padian K (2008b). The Early Jurassic pterosaur *Dorygnathus banthensis* (Theodori, 1830). Special Papers in Palaeontology.

[ref-35] Paganoni A (2003). Eudimorphodon after 30 years, history of the finding and perspectives. Rivista del Museo Civico di Scienze Naturali “Enrico Caffi”.

[ref-36] Tischlinger H, Frey E (2013). A new pterosaur with mosaic characters of basal and pterodactyloid pterosauria from the Upper Kimmeridgian of Painten (Upper Palatinate, Germany). Archaeopteryx.

[ref-37] Unwin DM, Buffetaut E, Mazin JM (2003). On the phylogeny and evolutionary history of pterosaurs. Evolution and palaeonbiology of pterosaurs.

[ref-38] Vidovic SU, Martill DM, Hone DWE, Witton MP, Martill DM (2017). The taxonomy and phylogeny of *Diopecephalus kochi* (Wagner, 1837) and ‘*Germanodactylus rhamphastinus*’ (Wagner, 1851). New perspectives on pterosaur palaeobiology.

[ref-39] Vullo R, Marugán-Lobón J, Kellner AWA, Buscalioni ÁD, Gomez B, De la Fuente M, Moratalla JJ (2012). A new crested pterosaur from the Early Cretaceous of Spain: the first European tapejarid (Pterodactyloidea: Azhdarchoidea). PLOS ONE.

[ref-40] Wang X, Jiang S, Zhang J, Cheng X, Yu X, Li Y, Wei G, Wang X (2017). New evidence from China for the nature of the pterosaur evolutionary transition. Scientific Reports.

[ref-41] Wang X, Kellner AWA, Jiang S, Cheng X (2012). New toothed flying reptile from Asia: close similarities between early Cretaceous pterosaur faunas from China and Brazil. Naturwissenschaften.

[ref-42] Wang X, Kellner AWA, Jiang S, Meng X (2009). An unusual long-tailed pterosaur with elongated neck from western Liaoning of China. Anais da Academia Brasileira de Ciencias.

[ref-43] Wang X, Kellner AWA, Zhou Z, Campos DA (2005). Pterosaur diversity and faunal turnover in Cretaceous terrestrial ecosystems in China. Nature.

[ref-44] Wang X, Kellner AWA, Zhou Z, Campos DA (2007). A new pterosaur (Ctenochasmatidae, Archaeopterodactyloidea) from the Lower Cretaceous Yixian Formation of China. Cretaceous Research.

[ref-45] Wang X, Lü J (2001). Discovery of a pterodactylid pterosaur from the Yixian Formation of western Liaoning, China. Chinese Science Bulletin.

[ref-46] Wang X, Rodrigues T, Jiang S, Cheng X, Kellner AWA (2014). An Early Cretaceous pterosaur with an unusual mandibular crest from China and a potential novel feeding strategy. Scientific Reports.

[ref-47] Wang X, Zhou Z (2004). Pterosaur embryo from the Early Cretaceous. Nature.

[ref-48] Wellnhofer P (1970). Die Pterodactyloidea (Pterosauria) der Oberjura-Plattenkalke süddeutschlands, Bayer. Akademie der Wissenschaften, Mathematisch-Naturwissenschaftliche Klasse, Abhandlungen, Neue folge Heft.

[ref-49] Wellnhofer P (1975). Die Rhamphorhynchoidea (Pterosauria) der Oberjura-Plattenkalke süddeutschlands Teil I. Palaeontographica Abteilung A.

[ref-50] Wellnhofer P (1978). Pterosauria. Handbuch der paläoherpetologie Teil.

[ref-51] Wellnhofer P (1985). Neue Pterosaurier aus der Santana-Formation (Apt) der Chapada do Araripe, Brasilien. Palaeontographica Abteilung A.

[ref-52] Wellnhofer P, Kellner AWA (1991). The skull of *Tapejara wellnhoferi* Kellner (Reptilia, Pterosauria) from the Lower Cretaceous Santana Formation of the Araripe Basin, Northeastern Brazil. Mitteilungen der Bayerischen Staatssammlung für Paläontologie und Historische Geologie.

[ref-53] Williston SW (1902). On the Skull of *Nyctodactylus*, an Upper Cretaceous Pterodactyl. Journal of Geology.

[ref-54] Williston SW (1903). On the osteology of *Nyctosaurus* (*Nyctodactylus*), with notes on American pterosaurs. Field Columbian Museum, Gelogical Series.

[ref-55] Witton MP (2012). New insights into the skull of *Istiodactylus latidens* (Ornithocheiroidea, Pterodactyloidea). PLOS ONE.

[ref-56] Witton MP (2013). Pterosaurs: natural history, evolution, anatomy.

[ref-57] Zambelli R (1973). *Eudimorphodon ranzii* gen. nov., sp. nov., uno pterosauro triassico. Rendiconti/Istituto lombardo B, Scienze biologiche e mediche.

[ref-58] Zhou Z, Zhang F (2004). A precocial avian embryo from the Lower Cretaceous of China. Science.

